# Integral Drug-Device Combinations and Medical Device Regulation Article 117: The European Medicines Agency Perspective

**DOI:** 10.1007/s43441-026-00983-3

**Published:** 2026-08-01

**Authors:** Pascal Venneugues, Christelle Bouygues, Thomas Girard, Veronika Jekerle

**Affiliations:** 1 European Medicines Agency, Domenico Scarlattilaan 6, 1083 HS Amsterdam, The Netherlands; 2 Human Medicines Division, Pharmaceutical Quality, Amsterdam, The Netherlands; 3 Human Medicines Division, Regulatory Affairs, Amsterdam, The Netherlands

**Keywords:** Medical device regulation, Integral drug-device combination, Article 117, Notified body opinion

## Abstract

Integral drug-device combinations (iDDCs) are governed in the European Union (EU) under the medicinal products framework, but the medical device part must meet relevant requirements of the medical device framework. This implies increased complexity as actors with different responsibilities, expertise and priorities are involved. Regulation (EU) 2017/745 on medical devices (MDR) [1] came into effect on 26 May 2021. At the time of the repealed Council Directive 93/42/EEC on medical devices (MDD) [2], the European Medicines Agency (EMA) and EU National Competent Authorities (NCAs) were overseeing the evaluation of the quality, safety, efficacy, benefit/risk of the medicinal product and the relevant Essential Requirements according to the MDD of the integral medical device used in combination. Article 117 of the MDR introduced a fundamental change: notified bodies became responsible for assessing compliance of the integral medical device with the relevant General Safety and Performance Requirements (GSPRs), as set out in MDR Annex I. These GSPRs represent a more stringent set of requirements in comparison with the Essential Requirements in the MDD. One of the objectives of Article 117 and the MDR was to strengthen regulatory oversight of the device component of the iDDC and increase confidence in the regulatory system. Given the interface of the medicinal product and medical device legislations, the regulatory process for initial authorisation and lifecycle management of iDDCs is challenging to operate for pharmaceutical industry, EMA, EU NCAs and notified bodies. In particular, the implementation of Article 117 raised challenges to all stakeholders involved. In order to support implementation for pharmaceutical industry and EU medicines NCAs, EMA published dedicated guidance and engaged in various conferences and stakeholder meetings. EMA also facilitated discussion of issues with different actors and enhance mutual understanding and sharing of experience. Here, we present EMA’s perspective on Article 117 implementation challenges and opportunities to tackle them.

## Introduction

Approximately 20% of all marketing authorisation applications (MAAs) submitted to the European Medicines Agency (EMA) since 26 May 2021 concern medicinal products used in combination with an integral medical device, so-called ‘integral drug-device combinations’ (iDDCs) as defined in Regulation (EU) 2017/745 on medical devices (MDR). In the EU, iDDCs are regulated under the medicinal products framework. Most integral devices fall under the second sub-paragraph of Article 1(9) of the MDR. This means that such devices are intended to administer a medicinal product whose action is principal. The legislation defines an integral combination between the medicinal product and the medical device. These devices should form a single integral product together with the medicinal product at the time of placing on the market and not at the administration timepoint. It should be intended exclusively for use in the given combination and should not be reusable. The majority of iDDCs consist of prefilled syringes and prefilled pens/autoinjectors. Other examples of integral devices include certain inhalers, nasal sprays and more recently introduced on-body injectors with pre-loaded cartridge.

Article 117 of the new MDR requires marketing authorisation applicants to provide, for the device part of an iDDC, an EU declaration of conformity, a Notified Body Opinion (NBOp) or a relevant CE certificate issued by a notified body, as appropriate.

The implementation of Article 117 by EMA, EU national competent authorities (NCAs), notified bodies and pharmaceutical industry has been complex. Most issues have been related, directly or indirectly, to the NBOp requirement, with risk of delays in the approval of the concerned MAAs.

Here we report the EMA experience with the application of Article 117 to iDDCs and discuss challenges related to the timely availability of the NBOp and potential overlap in the review between EMA and notified bodies. We further present some potential solutions, for example increased alignment between EMA assessment of the MAA and NBOp, and lifecycle considerations to guide applicants of iDDCs so that such challenges can be overcome in the future.

## Challenges

### Applicability of Article 117

EMA observed that predictability in the qualification or classification of iDDCs and its components sometimes presented a challenge to applicants. Indeed, upon entry into effect of the MDR, EMA received many queries from pharmaceutical industry and EU regulators on this topic: e.g. integral medical device versus container closure system, integral versus co-packaged medical device, non-sterile and non-measuring Class I medical device versus higher risk Class device. These challenges are illustrated with two examples.

The first example concerns a prefilled syringe with a needle not yet attached to the commercialised finished product. In MAAs, EMA has observed inconsistent classifications for such prefilled syringes with a similar intended use ranging from non-sterile and non-measuring Class I, Class I sterile (Is) to Class IIa [3]. These translate into different regulatory requirements as only Class Is and Class IIa devices require a NBOp. For Class Is all relevant General Safety and Performance Requirements (GSPRs) need to be reviewed, not just sterility aspects (a similar principle applies to Class I measuring devices). In relation to Class I versus Class Is, EMA noticed that sterility in the context of the MDR is differently understood by stakeholders, with for example (1) sterility of the empty integral medical device shipped by the supplier to the drug product manufacturer; (2) sterility of the integral medical device at the time of placing the medicinal product on the market, with its own packaging to maintain sterility until administration; (3) sterility of the content of the iDDC [4].

The second example concerns the status of a medicinal product in a vial co-packaged with a prefilled syringe containing a solvent for reconstitution. It could be understood that such prefilled syringe, without a measuring function, would not qualify as a medical device if its intended purpose is limited to the reconstitution of the medicinal product. However, if such prefilled syringe is also intended to administer the reconstituted medicinal product, this could be considered an integral medical device and therefore Article 117 would apply.

Whilst medical device qualification and classification fall outside EMA’s legal remit, it can have a direct impact on the regulatory requirements and therefore on the evaluation of the MAA. Guidance from the Medical Device Coordination Group (MDCG) is available for borderline cases [5] However, updating this guidance on a regular basis to address challenges faced and experience gained by the regulators and stakeholders would be of value to enhance harmonisation and clarity. To increase awareness for stakeholders on potential challenges, EMA has published feedback on experience with concrete cases [6] and invite stakeholders to consult an NCA in case of doubt on the qualification of a product as a medical device or its risk class.

EMA also noted cases where the marketing authorisation applicants were unable to provide an EU declaration of conformity for non-sterile and non-measuring Class I integral medical devices. A reason is that the different device parts of the iDDC, often sourced from external suppliers, are assembled as part of the drug product manufacturing process under Good Manufacturing Practices (GMP). The pharmaceutical company in charge of these operations does not always have the status of medical device manufacturer and therefore is not always in a position to issue an EU declaration of conformity. Thus, to overcome this issue, marketing authorisation applicants have sometimes applied for NBOps. To avoid misuse of NBOps primarily foreseen for risk class devices requiring notified bodies involvement, EMA recently published a clarification that when an EU declaration of conformity is not available for non-sterile and non-measuring Class I integral medical devices, marketing authorisation applicants may instead submit a statement of compliance with the relevant GSPRs [6].

The issuing of an EU declaration of conformity or CE certificate for the medical device of an iDDC regulated under Article 117 has been challenged by various stakeholders. This would imply for example affixing a CE mark and assigning a unique device identifier (UDI) to the device part of the iDDC, which would be conflicting with the fact that iDDCs are regulated under the medicinal products framework. This would also imply establishing a contractual agreement with notified bodies for the lifecycle management whilst it would not be the case for a NBOp. To date, EMA has not yet received a CE certificate to demonstrate compliance with Article 117, despite the significant number of iDDC products reviewed.

Due to the challenges described above and below for marketing authorisation applicants, EMA has observed that for a number of MAAs submitted since 26 May 2021, concerning medicinal products which could have been presented as ready-to -use iDDCs, alternative non-integral presentations within the meaning of Article 117, such as co-packaged devices or devices supplied separately, were proposed instead. This was observed even if in some cases no technical barriers to the development of a more convenient, fully assembled and ready-to-use iDDC were identified. A typical example is a vial containing the medicinal product to be administered with a separately obtained syringe, requiring several preparation steps and storage of the prepared syringe, sometimes for a long time, until administration of the medicinal product. Another example is a prefilled syringe without staked needle at the time of placing the medicinal product on the market, resulting sometimes in the observed downgrading of the device risk class from Class IIa (NBOp required) to non-sterile and non-measuring Class I (NBOp not required). EMA emphasises that ready-to-use iDDC should be developed whenever possible to minimise the risks inherent to the reconstitution and assembly of the combination product.

### Timely Availability of the NBOp

The MDR does not specify timelines for the notified body review and issuing of the NBOp. However, MAAs are subject to defined timelines [7] and demonstration of full compliance with Article 117 is required before the EMA’s Committee for Human Medicinal Products (CHMP) can adopt a positive opinion on the approval of the iDDC [6]. It is essential to have the NBOp stating compliance with the relevant GSPRs on time to prevent any delay in the authorisation of the iDDC.

From cases submitted to EMA, when the NBOp concluded only on partial GSPR compliance, i.e. deficiencies identified by the notified body in relation to relevant GSPRs in Chapters I, II and III of MDR Annex I, the marketing authorisation applicant needs to liaise with the notified body to resolve the issues and provide a revised NBOp confirming full compliance with the relevant GSPRs.

When the intended use of the iDDC is not congruent with the NBOp, EMA/CHMP raises objections during the MAA review, e.g. a measuring function required for administration but not covered in the NBOp.

EMA recommends applicants to provide the NBOp at the time of initial MAA submission in order not to delay the CHMP opinion. Nevertheless, EMA’s experience showed that the NBOp tends to be provided during the MAA procedure (e.g. at the time of responses to the CHMP list of questions or list of outstanding issues). Absence of the NBOp at MAA submission may be due to several factors beyond the applicant’s full control. For example, notified body capacity has been a recurrent concern [8, 9] along with their limited experience with this new procedure. EMA also observed cases where the marketing authorisation applicant was forced to change the notified body during the MAA procedure because the first one chosen was unable to issue a NBOp after a significant amount of time, sometimes longer than 12 months. Experience shared by notified bodies, on the other hand, also indicated that applicants sometimes provided them with inadequate technical documentation with missing or not sufficiently structured information, thereby causing delays in their reviews. Certain notified bodies published best practice guidelines to support applicants in preparing their NBOp applications [10].

EMA emphasises to marketing authorisation applicants that advance planning is critical to reduce the risk of delays in obtaining the NBOp. Aspects to consider include the amount of time needed to engage with a notified body, and prepare the required documentation for the NBOp application, and the notified body review time, currently understood to be around 6 months on average [11].

### Perceived Duplication of Review Between CHMP/NCAs and Notified Bodies

EMA’s guideline on quality documentation for medicinal products specifies the expected MAA dossier requirements for medicinal products used in combination with an integral or co-packaged medical device, or a medical device referenced in the product information of the medicinal product [12]. When a medicinal product is used with a medical device, the guideline clarifies the interface of assessment between EMA and the notified bodies i.e. EMA, or the relevant EU NCA responsible for the regulation of medicines, will evaluate the device (part) specific aspects relevant to the quality, safety and efficacy (and hence overall benefit/risk determination) of the medicinal product and the notified body will assess the relevant GSPRs for the device (part).

It implies that for a NBOp application, scope and outcome of the notified body review fall outside EMA’s remit. And quality, safety and efficacy of the iDDC fall outside the remit of notified bodies. However, in practice, some data, such as compatibility of the integral medical device with the drug product, certain functionality aspects or usability, are reviewed by both EMA/NCA and the notified body. For such data, which are linked to the clinical use of the medicine, EMA cannot just rely on the notified body review but requires the corresponding information in the MAA dossier and evaluate it in the context of the iDDC, with a different scope than the notified body review that is solely focused on the device part. For example, functionality is typically expected to be reflected in the drug product release specifications in the MAA dossier, with a justification on the suitability of the tests and acceptance criteria. Overall, it is important to appreciate any perceived duplication in the light of the objective of the review performed by the EMA/NCA and the notified bodies bringing a different perspective.

While an IDDc is regulated as a medicinal product, compliance with the GSPRs does not fall within the remit of the medicine regulators. EMA initially received NBOps with partial GSPR compliance issues on matters considered to fall within the remit of EMA/CHMP, for example transport validation or full real-time stability data to cover the shelf life of the iDDC. For shelf life, marketing authorisation applicants provide additional long-term stability data during the MAA procedure in order not to delay the MAA submission. Therefore, EMA advised that up-to-date stability data are not expected to be available at the time of NBOp application, especially if the objective is to have the NBOp available for the initial MAA submission. While there can be a difference in the extent of the data available at the time of NBOp and the MAA, it is important that the NBOp clearly describes the reviewed data to facilitate the downstream review by EMA/NCA during the MAA.

### Post-authorisation Lifecycle Management

**Fig. 1:**
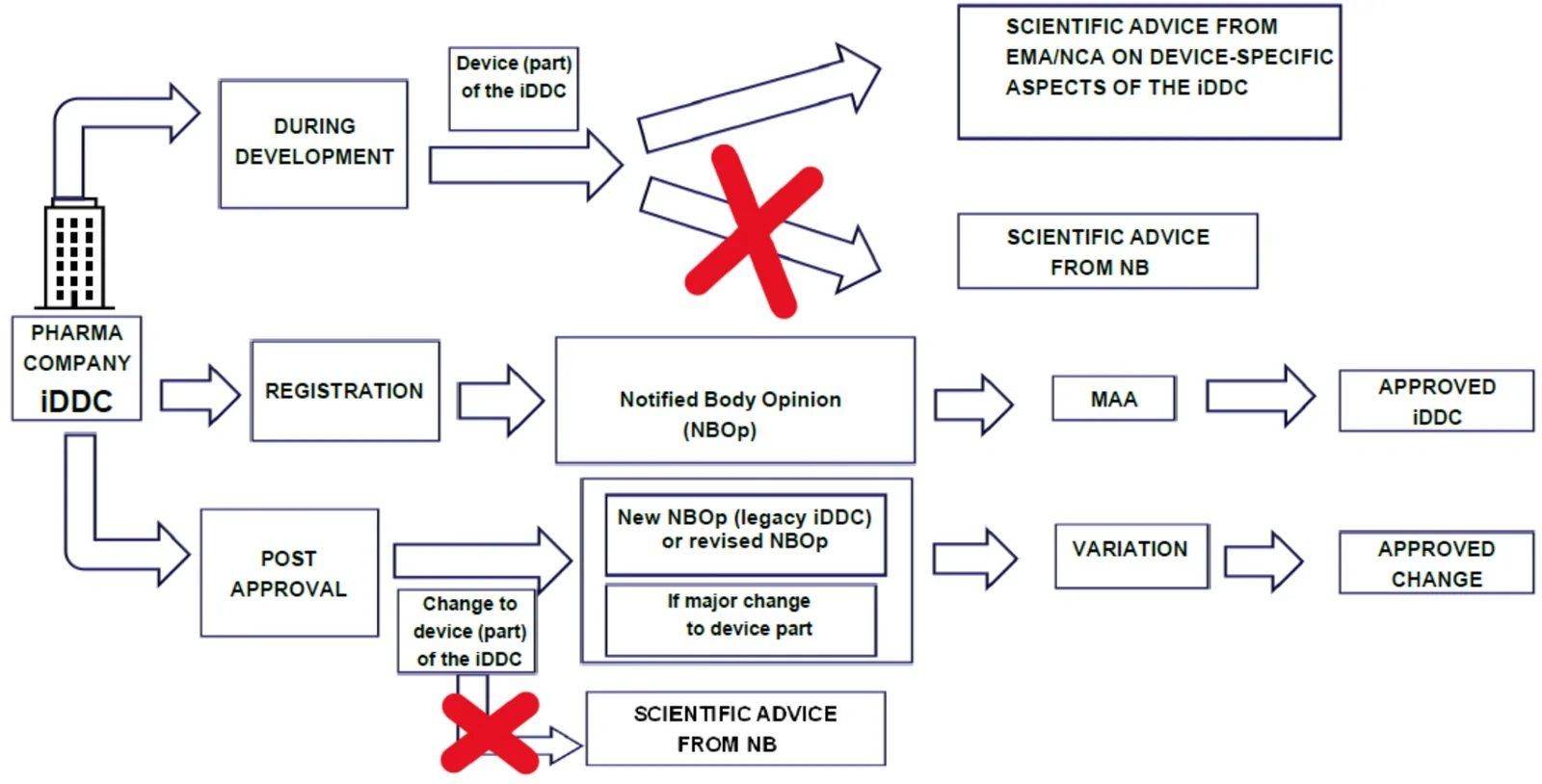
Lifecycle of integral drug-device combination (iDDC) subject to Notified Body Opinion (NBOp)

Although Article 117 does not contemplate changes to an authorised iDDC, they are pivotal for the lifecycle management of the iDDC, irrespective of whether it was authorised under the MDD or the MDR. (Fig. 1). Legacy iDDCs (MAA submitted before 26 May 2021) may be impacted by Article 117 requirements on an ad hoc basis. Depending on post-authorisation changes, a new NBOp may be required in case a new integral device is introduced, or a major change is made to the authorised integral device.

It is essential for stakeholders to have predictability on what constitutes a major change to an authorised integral medical device. This conditions whether a new or revised NBOp is required. EMA has been faced with situations where clarity could be improved in this regard.

In general, EMA’s experience suggests that major changes to an integral medical device concern its design, safety and performance characteristics, or its intended use. In addition, the impact on the quality target product profile, product critical quality attributes, control strategy, delivery, instructions for use should also be considered.

Whilst no definition for a major change to a medical device is contemplated in the MDR, some guidance has been developed in this regard, namely MDCG 2020-3 on significant changes [13], ISO standard 20,069 regarding assessment and evaluation of changes to drug delivery systems and TEAM-NB position paper on integral device-related changes [14]. As these documents have been developed to support the lifecycle of the medical device part solely, they have limited relevance for EMA and EU NCAs when evaluating the impact of the device change(s) on the quality, safety and efficacy of the iDDC. For example, the introduction of a new paediatric indication is considered by notified bodies as a substantial change in intended purpose, which means that a new or revised NBOp should systematically be obtained [14]. However, EMA considers that in case the device part, the intended user (e.g. healthcare professionals) and the instructions for use remain unchanged, and no new usability study is needed, a new or revised NBOp may not be required. Another example concerns the introduction of a new medical device manufacturer when the device material and specifications remain the same or similar. Whilst such a change would imply a new medical device and therefore systematically require a new or revised NBOp, regardless of the similarities or differences between the two devices, EMA considers that a new or revised NBOp may not be required if the device-related differences between old and new manufacturer are not major.

In relation to post-authorisation lifecycle management for iDDCs, EMA advises on changes requiring either a variation or an extension of marketing authorisation [15]. A difficulty faced by stakeholders is that notified bodies cannot provide advice on device-related changes and their impact on the NBOp, even when they already issued a NBOp for a particular iDDC (Fig. 1). Provision of regulatory or scientific advice is excluded from the legal mandate of notified bodies. Beyond that fact, contrary to a CE certificate, which is subject to contractual arrangements between the notified body and the device manufacturer for its lifecycle management, the NBOp is a one-off assessment without contractual obligations for the manufacturer to notify the notified body on the lifecycle management.

EMA observes that in defining whether a change to a medical device is substantial or not, the potential impact on safety or performance of the device against relevant GSPRs is considered by the operators and notified bodies. Whilst this determination falls outside EMA’s remit, it is important to highlight that for the purpose of iDDC, such change to the medical device can be evaluated in isolation; but the potential impact on quality, safety and efficacy of the medicinal product needs to be taken into account by the EMA/NCA.

Consequently, considering all these aspects, EMA has supported and advised applicants, on a case-by-case basis and following a risk-based approach, on integral device changes considered to require, or not, a new or revised NBOp.

### Discrepancies Between NBOp and EU Declaration of Conformity / CE Certificate

In relation to the documentation to establish conformity with the relevant GSPR *as per* Article 117, the level of requirements differs between a NBOp and a CE certificate or EU declaration of conformity. The NBOp is currently considered a one-off transaction between the applicant, which does not have to be a medical device manufacturer, and the notified body; and it is limited to compliance with the relevant GSPRs. On the other hand, the EU declaration of conformity and CE certificate can only be issued to a medical device manufacturer and in this case all applicable obligations and requirements of the MDR should be fulfilled, taking into account the device risk class, including the quality management system, audits, and ongoing maintenance of the technical documentation. Furthermore, there is no extended relationship foreseen between operators and notified bodies and no requirement or expectation for ongoing maintenance of the NBOp. Whilst for the most commonly encountered integral devices (e.g. syringe), regulators can assess the impact of the device changes directly in the context of the variation procedure, a more regular involvement of notified bodies in the lifecycle management of complex medical devices, for example on-body delivery systems (OBDS) may be needed. Alignment in the requirements with CE marked devices should be considered. It is indeed difficult to justify different device-related requirements between for example an OBDS with a pre-loaded cartridge (iDDC subject to NBOp) and the same or similar OBDS co-packaged with the cartridge at the time of placing the medicinal product on the market (co-packaged medical device subject to CE marking).

## Opportunities

### Platform Concept Considerations

There are some reflections whether a platform concept would be of relevance to be developed in order to leverage prior knowledge on a technology for medical devices to subsequent medicinal products incorporating a medical device part relying on this technology.

Independently from these reflections, it was questioned whether the same NBOp for a particular medical device could be used for different medicinal products. Whilst EMA would not have a priori objection, it would be important to determine if what is covered in the NBOp is appropriate and sufficient to address the use of the same medical device with a different medicinal product. It is expected that some data would be product-specific, for example different viscosity of the drug product formulation which could impact device performance, compatibility issues, or a different intended use(r). If product-specific aspects significantly impact the medical device, a new NBOp would be required.

### Opportunities for Closer Interactions Between EMA and Notified Bodies

The EMA Regulatory Science Strategy to 2025 [16] was published in 2020 as a plan for advancing EMA’s engagement with regulatory science over these five years. In relation to medical devices, it considered the development of an integrated evaluation pathway for the assessment of combination products; and one of the recommendations was the creation of a process to consult medical device authorities and/or notified bodies for device-related aspects throughout the product lifecycle. Work started to enhance collaboration between the various actors involved in combination products; this effort will be maintained as part of the network strategy to 2028 [17].

As an example of interactions, ad hoc meetings have been held between EMA and the Coordination Group of Notified Bodies for medical devices (NBCG-Med) which was established with the MDR. The objectives of these meetings were to exchange on the experiences and perspectives from medicine regulators and notified bodies on the application of Article 117 and to discuss improvements that could be achieved within the current regulatory framework. Another example of interaction was a meeting organised between EMA and the MDCG working group on borderline and classification to exchange on experience related to various device qualification and classification issues. A last example of interactions was meetings organised between EMA and representatives of the Coordination Group for Mutual Recognition and Decentralised procedures – Human (CMDh) with the aim of clarifying and harmonising the handling of the medical device issues in the context of iDDCs across EMA and EU NCAs.

EMA considers that iDDCs would benefit from an established framework for interactions with device bodies including notified bodies throughout the product lifecycle, from development and MAA pre-submission to MAA evaluation and post-authorisation. EMA should ensure involvement of the relevant expertise. This would be of particular relevance for complex integral medical devices, for example OBDS, or integral medical devices requiring specific expertise and considerations (e.g. tablets containing a medicinal product with an embedded sensor to monitor adherence to treatment). Increased interactions with relevant MDCG working groups would also be beneficial for seeking medical authorities’ input on issues regarding device aspects falling outside EMA’s legal remit.

In October 2025, EMA and the European Commission established an operational group on combination products, referred to as COMBO [18]. This group brings together experts from the EU NCAs for medicines and medical devices, NBCG-Med, EMA and the European Commission. The objectives are to facilitate dialogue, promote shared understanding and collaboratively explore potential regulatory solutions for medicines used in combination with medical devices. The NBOp has been identified as a priority topic with issues such as potential overlaps in the assessment performed by CHMP and notified bodies, inconsistencies between NBOps, insufficient data provided by applicants, inconsistent documentation expectations, timely availability of the NBOp. Work is currently underway to explore potential ways forward to streamline NBOp scope and assessment to enhance its complementarity with the regulators’ assessment of the quality module of the marketing authorisation application.

### Risk-Proportionate Approach

Given the extensive experience and expertise from EMA/CHMP, which dates back to the Council Directive 93/42/EEC on medical devices (MDD), where EMA/CHMP oversaw the full evaluation including compliance with MDD essential requirements without quality, safety and efficacy issues emerging from this, and acknowledging the need of increasing efficiency and EU competitiveness, a risk-proportionate approach on the need to provide a NBOp in support of medical device part of iDDC could be considered. This would allow an optimised management of the available expertise and capacity of notified bodies that is particularly required for complex or novel cases, whilst less relevant for standard non-complex cases.

The need for a NBOp could be based on agreed criteria and on the impact on quality, safety and efficacy of the iDDC. Regulators could provide guidance on such risk criteria, for example device novelty or complexity. This approach would address various issues including duplication in the submission of data to both the notified body and EMA, notified body capacity, timely approval of MAAs, and decrease of regulatory burden for marketing authorisation applicants. This could be considered by analogy to what is already set out in Regulation (EC) No 1394/2007 on advanced therapy medicinal products (ATMPs) [19]. Involvement of a notified body for a combined ATMP is foreseen in Article 9 of the ATMP Regulation. Differing from Article 117, this involvement is not systematic but where needed for the evaluation of the ATMP, upon request of the EMA’s Committee for Advanced Therapies (CAT).

## Conclusion

EMA and stakeholders have faced significant challenges in applying Article 117 to the pre-and post-authorisation of medicinal products, including the lack of clarity in requirements, the timely availability of NBOps, and the perceived duplication of reviews between EMA/CHMP and notified bodies. A framework for closer interactions between relevant stakeholders is in our view essential to overcome these challenges. We also propose to consider the possibility of applying a risk proportionate approach, based on clearly defined criteria, in order to determine when a NBOp is required.

The EU legal framework has been developed to ensure patient safety and trust in the regulatory system whilst supporting innovation and timely access to medicines. Adaptations are necessary as science and experience evolve. In December 2024, the European Commission launched a public call for evidence [20] in preparation for an expected targeted revision of the MDR to ensure the legal framework continue to adapt to the new challenges encountered and fulfil the above-mentioned objectives. Following this public consultation, in December 2025 the European Commission published a proposal for amendments to the Regulation to reduce and simplify rules on medical devices [21].

In addition, the new EU pharma legislation has just been agreed [22] and opens up opportunities for improvement, such as the possibility for EMA to interact with device bodies for medical devices during the MAA assessment and to provide parallel and joint Scientific Advice on the development of medicinal products used in combination with medical devices. Such legislative changes, as well as lessons from accumulated experience with the MDR, and increased alignment and communication between stakeholders of the medicinal product and medical device frameworks offer a valuable opportunity to address issues related to Article 117 and help stakeholders and regulators overcome challenges in the approval and supervision of iDDCs in the EU.

## Data Availability

No datasets were generated or analysed during the current study.

## References

[1] Regulation (EU). 2017/745 of the European Parliament and of the Council of 5 April 2017 on medical devices. Available online: https://eur-lex.europa.eu/legal-content/EN/TXT/?uri=CELEX%3A02017R0745-20250110

[2] Council Directive 93/42/EEC of 14. June 1993 concerning medical devices. Available online: https://eur-lex.europa.eu/eli/dir/1993/42/2007-10-11

[3] MDCG 2021-24 Guidance on classification of medical devices. Available online: https://health.ec.europa.eu/system/files/2021-10/mdcg_2021-24_en_0.pdf

[4] CHMP Guideline on the sterilisation of the. medicinal product, active substance, excipient and primary container. Available online: www.ema.europa.eu/en/documents/scientific-guideline/guideline-sterilisation-medicinal-product-active-substance-excipient-and-primary-container_en.pdf

[5] MDCG 2022-5 Rev. 1 Guidance on borderline between medical devices and medicinal products under Regulation (EU) 2017/745 on medical devices. Available online: https://health.ec.europa.eu/latest-updates/mdcg-2022-5-rev1-guidance-borderline-between-medical-devices-and-medicinal-products-under-regulation-2024-10-29_en

[6] Questions. & Answers for applicants, marketing authorisation holders of medicinal products and notified bodies with respect to the implementation of the Regulations on medical devices and in vitro diagnostic medical devices (Regulations (EU) 2017/745 and (EU) 2017/746). Available online: www.ema.europa.eu/en/documents/regulatory-procedural-guideline/questions-answers-implementation-medical-devices-vitro-diagnostic-medical-devices-regulations-eu-2017-745-eu-2017-746_en.pdf

[7] European Medicines Agency. 2024. The evaluation of medicines, step-by-step. Available online: https://www.ema.europa.eu/en/human-regulatory-overview/marketing-authorisation/evaluation-medicines-step-step

[8] MDCG 2022-14 Position Paper on Transition to the MDR and IVDR, Notified body capacity and availability of medical devices and IVDs. (2022). Available online: https://health.ec.europa.eu/system/files/2022-08/mdcg_2022-14_en.pdf

[9] TEAM-NB Position Paper New MDR Transition Timelines and Notified Body Capacity. 2023. Available online: https://www.team-nb.org/wp-content/uploads/2023/08/Team-NB-PositionPaper-MDRTransitionTimelines-NotifiedBodyCapacity-V1.pdf

[10] Article. 117 - Documentation submission best practice guidelines (BSI). Available online: www.bsigroup.com/en-GB/insights-and-media/insights/brochures/article-117-documentation-submission-guidelines/

[11] Position Paper on Navigating EU MDR Article 117, Industry Experience EFPIA. June 2025. Available online: https://www.efpia.eu/media/jv0e1w5u/efpia-publication-draft-art-117_final-june-2025.pdf

[12] CHMP guideline on quality documentation for medicinal products when used with a medical device. Available online: www.ema.europa.eu/en/documents/scientific-guideline/guideline-quality-documentation-medicinal-products-when-used-medical-device-first-version_en.pdf

[13] MDCG 2020-3. Guidance on significant changes regarding the transitional provision under Article 120 of the MDR with regard to devices covered by certificates according to MDD or AIMDD Rev.1 Available online: https://health.ec.europa.eu/system/files/2023-09/mdcg_2020-3_en_1.pdf

[14] TEAM-NB Position paper for the interpretation of. device related changes in relation to a Notified Body Opinion as required under Article 117 of Medical Device Regulation (EU)2017/745. Available online: www.team-nb.org/wp-content/uploads/2020/12/Team-NBPosition-Paper-Art117SubChangeLifeCycleMngt-202012.pdf

[15] EMA post-authorisation guidance. Available online: www.ema.europa.eu/en/human-regulatory-overview/post-authorisation/contacting-ema-post-authorisation

[16] EMA Regulatory Science. to 2025 - Strategic reflection. Available online: www.ema.europa.eu/en/documents/regulatory-procedural-guideline/ema-regulatory-science-2025-strategic-reflection_en.pdf

[17] European medicines agencies network strategy to 2028. Available online: https://www.ema.europa.eu/en/about-us/how-we-work/european-medicines-regulatory-network/european-medicines-agencies-network-strategy

[18] Combination Products Operational Group (COMBO) – Terms of reference. Available online: https://www.ema.europa.eu/en/documents/other/combination-products-operational-group-combo-terms-reference_en.pdf

[19] Regulation (EC). No 1394/2007 of the European Parliament and of the Council of 13 November 2007 on advanced therapy medicinal products. Available online: https://eur-lex.europa.eu/legal-content/EN/TXT/PDF/?uri=CELEX:32007R1394

[20] EU rules on medical devices and in vitro diagnostics – targeted evaluation. Available online: https://ec.europa.eu/info/law/better-regulation/have-your-say/initiatives/14155-EU-rules-on-medical-devices-and-in-vitro-diagnostics-targeted-evaluation_en

[21] Proposal for a Regulation of the European Parliament and the Council amending Regulations (EU) 2017/745 and 2017/746. Available online: https://health.ec.europa.eu/medical-devices-sector/new-regulations_en

[22] A pharmaceutical strategy for Europe. Available online: https://health.ec.europa.eu/medicinal-products/pharmaceutical-strategy-europe_en

